# Usefulness of FDG-PET/CT-Based Radiomics for the Characterization and Genetic Orientation of Pheochromocytomas Before Surgery

**DOI:** 10.3390/cancers12092424

**Published:** 2020-08-26

**Authors:** Catherine Ansquer, Delphine Drui, Eric Mirallié, Karine Renaudin-Autain, Antoine Denis, Anne-Paule Gimenez-Roqueplo, Christophe Leux, Frederique Toulgoat, Françoise Kraeber-Bodéré, Thomas Carlier

**Affiliations:** 1 Nuclear Medicine Department, University Hospital of Nantes, Place Alexis Ricordeau, 44093 Nantes, France; a.denis@ch-rodez.fr (A.D.); francoise.bodere@chu-nantes.fr (F.K.-B.); thomas.carlier@chu-nantes.fr (T.C.); 2 Université de Nantes, CNRS, INSERM, CRCINA, 44007 Nantes, France; 3 Endocrinology Department, University Hospital of Nantes, Boulevard Jacques Monod, 44092 Nantes, France; delphine.drui@chu-nantes.fr; 4 Digestive and Endocrine Surgery Department (CCDE), Institut des Maladies de l’Appareil Digestif (IMAD), University Hospital of Nantes, Place Alexis Ricordeau, 44093 Nantes, France; eric.mirallie@chu-nantes.fr; 5 Anatomopathology Department, University Hospital of Nantes, Place Alexis Ricordeau, 44093 Nantes, France; karine.renaudin@chu-nantes.fr; 6 Genetic Department, Assistance Publique Hôpitaux de Paris, Hôpital Européen Georges Pompidou, 75015 Paris, France; anne-paule.gimenez-roqueplo@aphp.fr; 7 Université de Paris, INSERM, PARCC, 75015 Paris, France; 8 Medical Information Department, University Hospital of Nantes, Place Alexis Ricordeau, 44093 Nantes, France; christophe.leux@chu-nantes.fr; 9 Radiology Department, University Hospital of Nantes, Place Alexis Ricordeau, 44093 Nantes, France; frederique.toulgoat@chu-nantes.fr

**Keywords:** pheochromocytoma, paraganglioma, 18FDG, PET/CT, radiomics, adrenal gland, germline mutation

## Abstract

**Simple Summary:**

Around 40% of patients with pheochromocytomas (PHEO) carry a germline mutation. Early germline mutation identification is important for accurate treatment and follow-up in affected patients. The aim of our retrospective study was to assess the potential added value of FDG-PET/CT radiomics for the characterization of PHEO and their genetic orientation prior to surgery and genetic testing. We confirmed in an homogeneous population of 52 PHEO (49 patients) the usual avidity of these tumors for FDG (92%) and the impact of germline mutation on their phenotypic presentations with higher SUVmax observed in Cluster-1-related genes. Radiomics biomarkers provided valuable additional and independent information for discriminating genetically determined PHEO (Cluster-1 or Cluster-2-related genes) as well as sporadic forms. FDG-PET/CT is then useful for preoperative detection of PHEO, and when combined with texture features, provides evidences for a genetic predisposition.

**Abstract:**

Purpose: To assess the potential added value of FDG-PET/CT radiomics for the characterization of pheochromocytomas (PHEO) and their genetic orientation prior to surgery and genetic testing. Methods: This retrospective monocentric study, included 49 patients (52 tumors) that underwent both FDG-PET/CT and MIBG scan before surgery. A germline mutation was secondarily identified in 13 patients in one of the genes related to Cluster 1 (*n* = 4) or Cluster 2 (*n* = 9). No mutation was identified in 32 patients and 4 did not have genetic testing. Correlation between several PET-based biomarkers, including SUVmax, metabolic tumor volume (MTV), total lesion glycolysis (TLG) and textural features, and biochemical and genetic features were analyzed. Results: Sensitivity of FDG-PET/CT alone was 92%, and 98% when combined to MIBG. The SUVmax was significantly higher for mutated tumors classified in Cluster 1 than in Cluster 2 (*p* = 0.002) or for tumors with no identified mutations (*p* = 0.04). MTV and TLG of the tumors with the most intense uptake discriminated mutated Cluster 2 from sporadic tumors, but not from Cluster 1 tumors. Textural features combined with MTV led to better differentiation between sporadic and mutated tumors (*p* < 0.05). Conclusion: FDG-PET/CT is useful for preoperative characterization of PHEO, and when combined with radiomics biomarkers, provides evidences for a genetic predisposition.

## 1. Introduction

Pheochromocytomas (PHEO) are rare neuroendocrine tumors arising from chromaffin cells of the adrenal glands. Most PHEO are revealed by clinical signs related to hyperproduction of catecholamines. The number of genetic syndromes predisposing to the occurrence of PHEO and the related extra-adrenal tumors called paragangliomas (PGL) is yet estimated to occur in about 40% of these patients. The most common genetic syndromes are Multiple Endocrine Neoplasia Multiple Type 2 (MEN 2, caused by RET gene mutations), Von Hippel Lindau Disease (VHL), Neurofibromatosis Type 1 (NF1) and SDHx-related PHEO/PGL. Tumors harboring mutations in SDHx or VHL genes are classified by transcriptomic analyses in a pseudo-hypoxic cluster called Cluster 1, whereas those with RET or NF1 are grouped into a kinase-signaling cluster called Cluster 2. The identification of a germline mutation exposes the patient to the risk of synchronous or metachronous multifocal disorders and, consequently, impacts the medical management. The risk of malignancy occurs in 15% of cases, but can reach 40% in patients with an SDHB mutation [1,2].

Metaiodobenzylguanidine (MIBG) is a norepinephrine analogue. When labeled with iodine 123 or 131, MIBG shows very high specificity to detect PHEO. However its use decreases due to its moderate sensitivity (75–85%) [3,4], especially in SDHx-related PHEO/PGL [5]. ^18^F-fluorodihydroxyphenylalanine (^18^F-FDOPA) PET/CT appears to be the most effective functional imaging modality for PHEO detection, with 84–100% sensitivity and 88–100% specificity [6,7,8]. However, its use worldwide is limited. ^18^F-fluorodeoxyglucose (^18^F-FDG) is the most common PET tracer used in nuclear medicine. It is highly recommended in the international guidelines for the workup of metastatic SDHB related PHEO/PGL [1,9] and FDG avidity is increased in the context of mutations in Cluster-1-related genes [10,11,12,13,14]. In PHEOs, a frequent but variable FDG-PET positivity is reported with a sensitivity and a negative predictive value of 80–100%. Nevertheless, the positive predictive value is lower due to lack of specificity of FDG and the sensitivity is also mainly influenced by the genetic status of the patient [10,11,15]. Despite this, the global usefulness of FDG-PET/CT in the preoperative characterization of PHEO would deserve to be more documented.

Radiomics is often described as the extraction of high-dimensional quantitative parameters from medical imaging [16]. This approach has now gained a significant interest in the medical community since several studies have reported its usefulness in the determination of molecular status, prediction of clinical outcome, prognosis, or response to treatment [17]. However, only a limited number of studies have focused on the potential benefit of using the textural features (TF) information in the context of adrenal tumors. One study used the CT-based TF information to differentiate PHEO from lipid-poor adrenocortical adenoma [18], and a second used FDG-PET based TF for discriminating benign from metastatic adrenal tumors [19].

Our objective was to retrospectively assess the impact of clinical, biological, histological and genetic parameters from FDG-PET/CT data recorded before surgery in patients with PHEO, and to assess the qualitative and quantitative analyses including metabolic tumor volume (MTV) and TF.

## 2. Results

### 2.1. Patient and Tumors Characteristics

The characteristics of the 49 patients and the 52 tumors studied are summarized in Table 1 and Table 2. Five patients had a family history of PHEO and germline mutations were identified in 13 patients: four patients with a mutation in one Cluster-1-related genes causing an activation of the hypoxic pathway (one VHL, one SDHD, one SDHB and one FH), and nine patients with a mutation in one Cluster-2-related gene activating the MAPK and mTOR signaling pathways (six RET and three NF1). No germline mutations were found in 32 patients (sporadic cases). Four patients refused the genetic test. Three patients had synchronous bilateral PHEO due to a RET mutation. Two patients had metastatic PHEO with bone metastases and no identified mutation. A patient with a VHL had synchronous thoracic PGL.

The tumor median size was 45 mm with 35 tumors ≥ 40 mm. Cluster-2-related tumors were significantly smaller than sporadic tumors (34.5 vs. 56.5 mm; *p* < 0.05) but were not significantly different from those of Cluster 1 (34.5 vs. 39.5 mm; *p* > 0.05). This could be explained by the early stage of detection of Cluster 2 due to other manifestations of the germline especially NF1 and then the smallest size of cluster 2 compared with sporadic tumours. Catecholamines excess was diagnosed in 44 patients (90%) and not determined for three patients before surgery. Predominant production of normetanephrine was observed in 2/4 patients of Cluster 1 (the two others had no secretion) and in 22/33 of non-mutated patients (nine had predominant secretion of metanephrine and two unknown secretion). Predominant secretion of metanephrine was present in 6/9 patients of Cluster 2 (two had predominant secretion of normetanephrine, and one had similar secretion of normetanephrine and metanephrine). The median PASS value was 4 [1–30] and the median Ki-67 index was 2 [0–18], with no significant difference associated with genetic status. The highest Ki-67 index was reported for the two patients with metastatic PHEO (at 25% and 30%, versus ≤ 5% for benign PHEO).

The values are median for continuous variable and exact number for categorical variable.

### 2.2. FDG-PET/ CT Analysis

Forty-eight tumors were FDG positive, and 39 tumors MIBG positive corresponding to a sensitivity of 92% and 75%, respectively. The combined sensitivity of FDG and MIBG procedures reached 98%. Of the 13 negative MIBG tumors, one partially necrotic lesion (53 mm) was FDG negative. The four FDG negative tumors had a median size of 33.5 mm [30–53]. One of them was observed in a patient with a single PHEO due to a RET mutation and the three others in patients with no identified mutation (including the only MIBG-/FDG-tumour).

**Figure 1:**
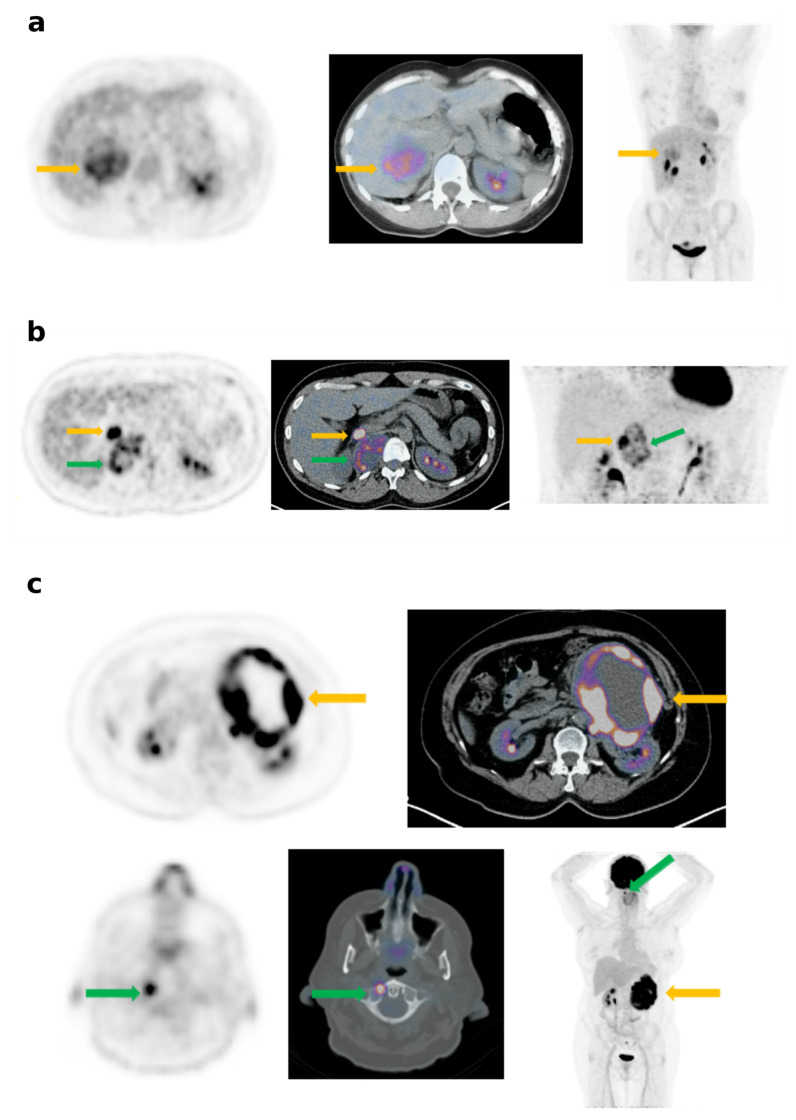
Different patterns of FDG uptake in PHEO: (**a**) Mild and heterogeneous uptake (SUVmax = 6.7; SUVmax lesion/liver ratio = 2.2) by a right sporadic PHEO of 60 mm (yellow arrow). (**b**) High uptake (SUV max = 21.2, SUVmax lesion/liver ratio = 5.1) by a right PHEO of 25 mm (yellow arrow) and a moderate and heterogeneous uptake by a thoracic PGL (green arrow) in a patient with a VHL disease. (**c**) High peripheric uptake (SUV max = 39.8, SUVmax lesion/liver ratio = 9.4) by a left metastatic PHEO of 180 mm (yellow arrow). The vertebral C1 metastasis exhibits a high FDG uptake (green arrow).

**Figure 2:**
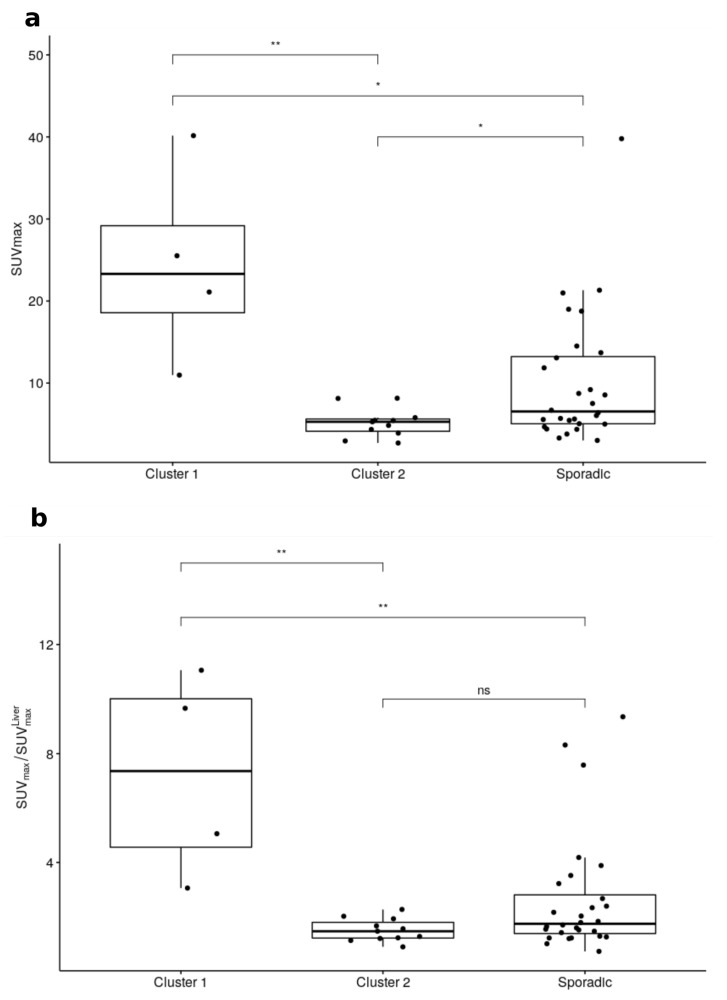
SUVmax (**a**) and ratio between SUVmax and liver SUVmax (**b**) according to genetic status (ns: non-significant, *: *p* < 0.05, **: *p* < 0.01).

**Figure 3:**
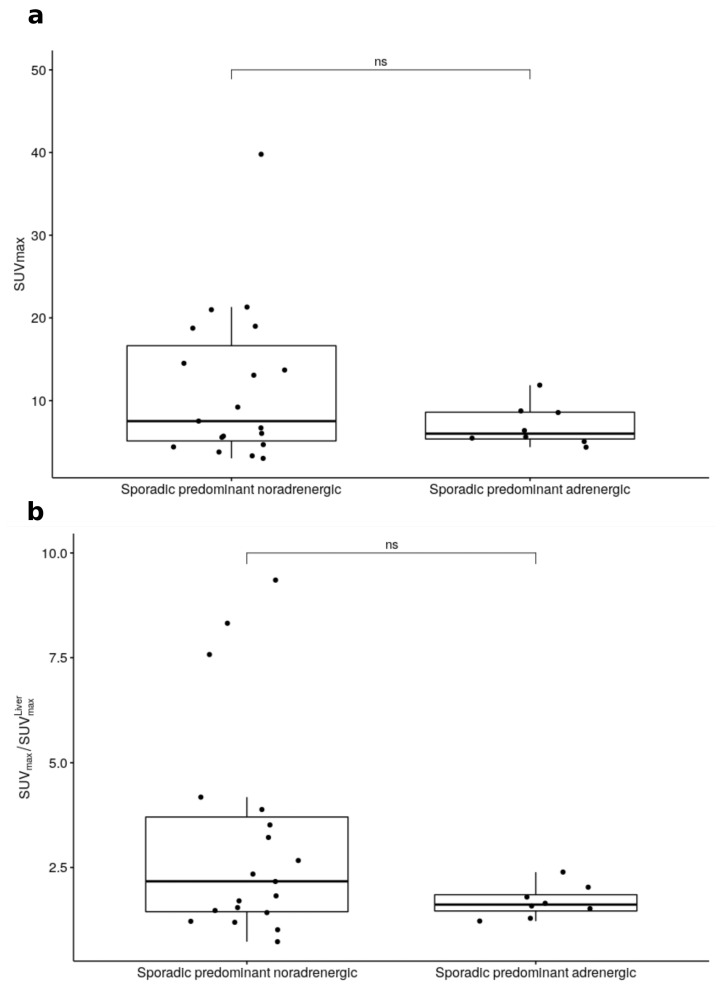
SUVmax (**a**) and ratio between SUVmax and liver SUVmax (**b**) according to predominant secretion for sporadic tumours (ns: non-significant).

PHEO demonstrated different patterns of FDG uptake as illustrated in Figure 1. The SUVmax was significantly higher (Figure 2) for Cluster 1 than for Cluster 2 or sporadic tumors, with a median SUVmax = 21.5 [11–40.3] versus 5.3 [2.5–8.2] for Cluster 2 (*p* = 0.002) and 6.5 [3.2–39.8] for sporadic (*p* = 0.04). Significant differences were observed between SUVmax of Cluster 2 and of sporadic PHEO (*p* = 0.03). Similar results were also observed for the adrenal tumor/liver SUVmax ratio (Table 2) except for the difference between Cluster 2 and sporadic tumors, which were non-significant in this case (Figure 2). Seven PHEO had a SUVmax > 15:3 with a mutation in Cluster-1-related genes (two SDHx and one VHL) and four sporadic, all of them with a predominant noradrenergic secretion, notably in a 13-year-old patient (SUVmax = 18.9) and in patient (SUVmax = 39.7) with a metastatic PHEO. Sporadic PHEO showed heterogeneity in terms of FDG uptake. In this group of tumours, no significant differences were found between SUVmax and adrenal tumour/liver SUVmax ratio according to the predominance of metanephrine secretion (Figure 3). Nevertheless, eight out of the nine sporadic PHEO with SUVmax above 10 were noradrenergic predominant secreting tumours. Additionally, while non-significant (*p* = 0.36), a general trend of higher SUVmax in sporadic predominant noradrenergic producing tumours (mean = 11.6) than in adrenergic producing tumours (mean = 7.0) was noted. It is not excluded that some of these patients with predominant noradrenergic producing tumours presented somatic cluster-1-related mutations. Conversely, patients classified as sporadic with low SUVmax and adrenergic predominant producing tumours are more likely belonging to cluster 2.

**Figure 4:**
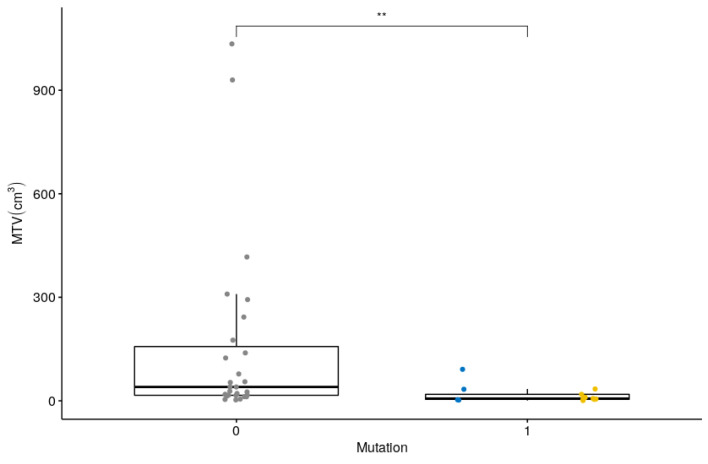
MTV according to the presence (1) or absence (0) of identified mutation (**: *p* < 0.01). Points in blue are for Cluster-1 and in yellow for Cluster-2-related genes.

**Figure A1:**
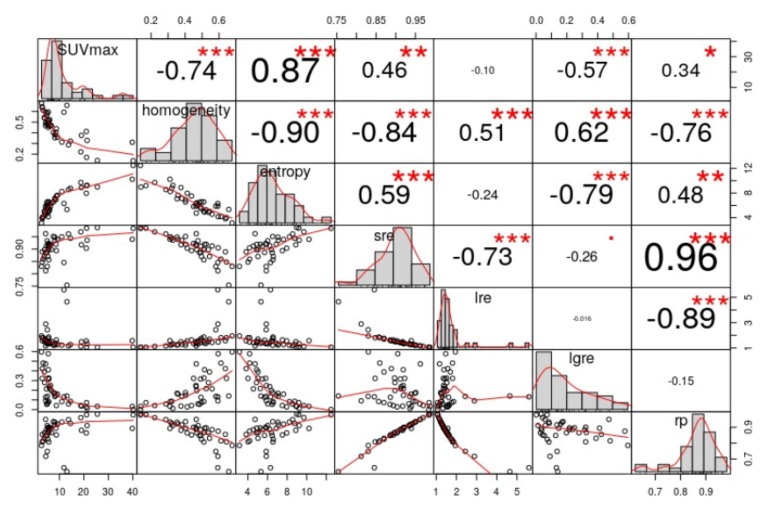
Intra-correlation of TF and SUVmax using absolute quantization equalization (*: *p* < 0.05, **: *p* < 0.01, ***: *p* < 0.001).

Most of the six TFs, chosen for being the most robust in the context of multiple PET systems and associated reconstruction settings and calculated using the absolute quantization, were also able to separate the three different genetic sub-groups. The TF that presented the lowest correlation with SUVmax (three TFs among the six, namely SRE, LRE and RP, Figure A1) did not outperform SUVmax in the ability to discriminate Cluster 1 from Cluster 2 or Cluster 1 from sporadic forms at ROC analysis. Only a few TF using the linear equalization were able to separate Cluster 1 or Cluster 2 from sporadic forms (HGRE and SZHGE) but none succeeded in discriminating Cluster 1 from Cluster 2. TMTV and wbTLG were not able to discriminate any of the three genetic sub-groups while MTV and TLG of the most intense adrenal lesion could discriminate Cluster 2 from sporadic forms (higher in sporadic forms). Patients that presented a mutation could be discriminated from a patient without mutation when considering MTV or TLG of the most intense lesion (Figure 4) with higher MTV and TLG in cases of sporadic forms than in mutated forms. It should be emphasized that MTV and tumor size as measured by histopathology were strongly correlated (Figure A4).

**Figure 5:**
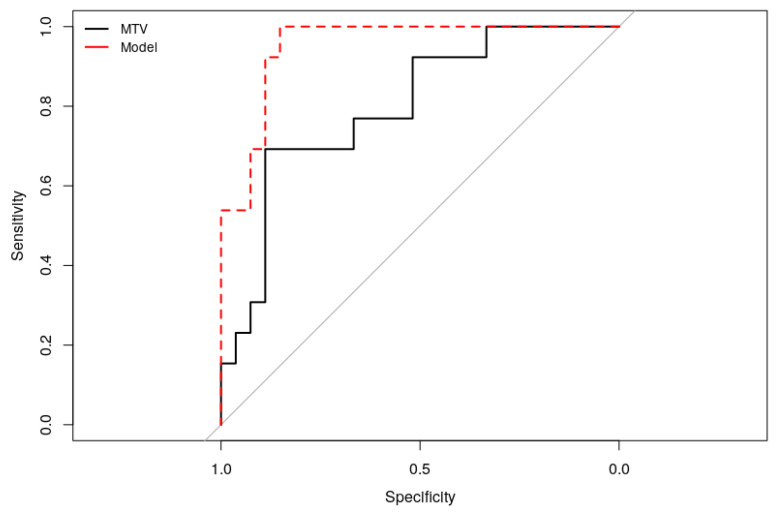
ROC curves for MTV and the model for discriminating patient with and without mutation.

**Figure A2:**
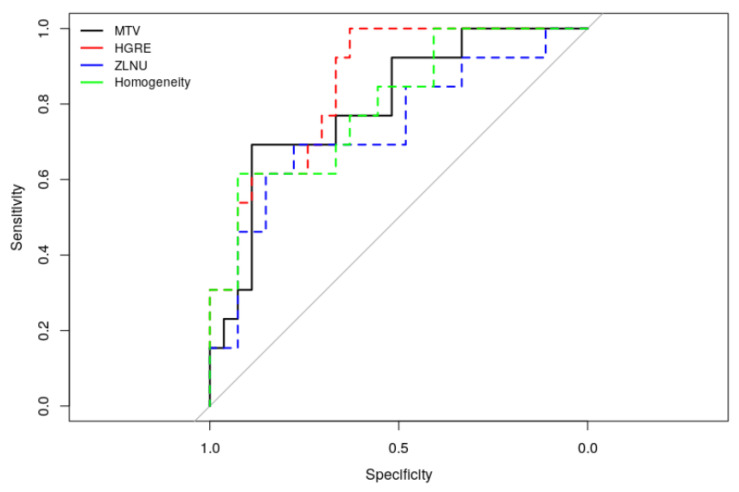
ROC curves for MTV and selected TF (one for each matrix) for discriminating patient with and without mutation.

**Figure A3:**
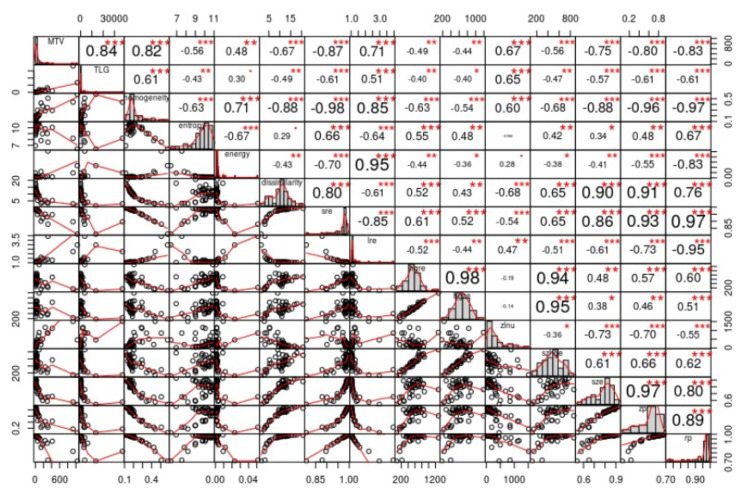
Intra-correlation of TF, MTV and TLG using linear equalization (*: *p* < 0.05, **: *p* < 0.01, ***: *p* < 0.001).

**Figure A4:**
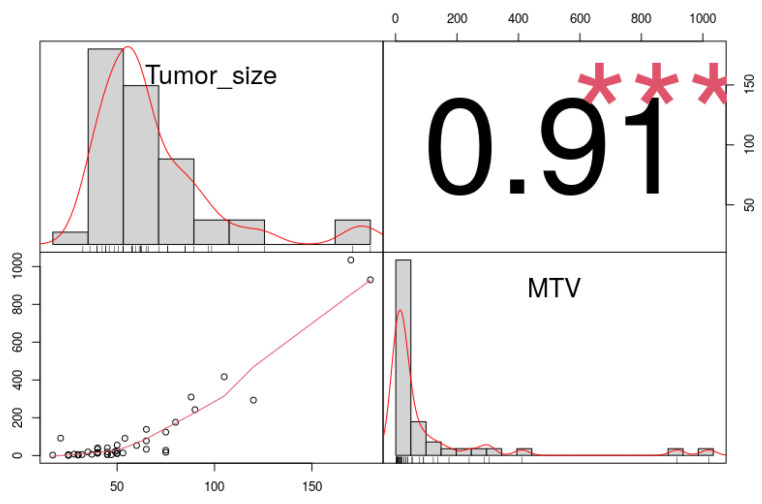
Correlation between tumor size and MTV (***: *p* < 0.001).

Using linear equalization, most TF performed equally in discriminating mutated forms from sporadic forms, but were not significantly different from MTV at the ROC analysis level (Figure A2). The multiple logistic regression model including TF that are not significantly correlated to MTV (Figure A3) markedly improved the separation of patients with and without mutations by selecting the MTV and two different TF (namely HGRE and ZLNU). The AUC for MTV was 0.78 vs. 0.95 for the model (*p* < 0.05). Figure 5 illustrates the ROC curves for the MTV alone and the model found in the multiple logistic regression.

The correlation analyses with PET parameters and TF were negative. In particular, no correlation was found between the semi-quantitative PET parameters and the PASS score or the Ki67 index. The predominant secretion was not significantly associated with SUVmax or any of the TF using the absolute quantization. This last result holds true for the different SUV ratios extracted. TMTV and wbTLG were not found to be associated to the secretion status.

## 3. Discussion

Because of their common embryologic origin, PHEO and PGL are associated. They are considered today to be the most heritable tumors with the number of susceptibility genes increasing each year (>15 genes identified). Because around 40% of these patients carry a germline mutation, PGL/PHEO genetic testing should be proposed to all affected patients [1]. Early germline mutation identification is important for accurate treatment and follow-up in affected patients and also for earlier diagnosis and treatment of PHEO/PGL of family members [20]. Genetic testing is today usually performed by NGS but the time delay before test results might be long. In the absence of a clinical or family context suggestive of a genetic predisposition, the early detection of a mutation in Cluster-1-related genes (including SDHB, for which a mutation is associated with a high risk of malignancy) by FDG-PET/CT could have an impact on post-operative monitoring, by modifying the timing and choice of imaging procedures.

FDG is the most widely used PET tracer for tumor explorations. The intensity of FDG uptake is considered as a prognostic biomarker in most endocrine tumors [15]. In adrenal pathology, the link between FDG uptake and the aggressiveness of tumors has not been correlated [11,21]. Nevertheless, FDG-PET/CT is validated (as well as ^68^Ga-DOTA-somatostatine analogs) for the diagnosis and assessment of the extent of metastatic PHEO/PGL and mutated SDHB [9,10]. Indeed, whilst most PHEO are FDG avid, there is currently no published large series that analyses PHEO populations exclusively.

Our study confirmed the high sensitivity of FDG-PET/CT (92%) in a homogeneous PHEO cohort and showed, as reported by Tiwari et al, that negative MIBG PHEO seemed to be mostly FDG positive. The phenotypic presentation differs according to genetic status. Sporadic PHEO had very moderate uptake and were larger whereas intense FDG uptake suggested the possibility of a mutation Cluster-1-related genes, particularly in young patients, multifocal PHEO/PGL, or in a context of PHEO/PGL family history. In our series, FDG avidity was significantly higher in the context of mutations in Cluster-1-related genes than in Cluster 2 or sporadic forms, as previously reported in PGL [10,11,12,13,14]. The FDG avidity of Cluster 1 lesions has been explained by a high expression of glucose transporters in SDHx- and VHL-related tumors [22].

Several authors pointed out the lack of standardization for computing TF together with very sparse indications related to the different options chosen among the various possibilities offered in the TF computation workflow [17]. One of the crucial steps involved in the computation is the choice of the discretization approach. We did not choose, a priori, a single discretization, since each method can bring different information (e.g., fixed bin number or fixed-width bins). However, we studied and chose only one parameter for each category since we wanted to use an implementation that has been already shown to be robust enough against technical perturbation based on results reported in the literature [23,24,25].

The use of TF in the context of PHEO is very sparse in the literature. One study reported results using CT imaging that was focused on TF based on histogram intensity, which is not strictly speaking equivalent to TF in the sense of spatial variation intensity [18]. A second study showed the potential of PET-based TF for discriminating benign from metastatic adrenal tumors using a very limited number of TF and too few details regarding their implementation [19]. As the objectives and the technical design were different, their work cannot be fully compared with this current study. Our study showed that TF provides valuable information for discriminating genetically determined PHEO (Cluster 1 and Cluster-2-related genes) from sporadic forms using a TF based on the fixed-width bin which is more prone to be correlated with SUVmax. However, these TF did not outperform results reported using SUVmax and ROC analysis therefore limiting their usefulness in this context. The benefit of using the TF information together with MTV was demonstrated for differentiating patients with and without mutations. Combining two TFs (ZLNU and HGRE) with the MTV enhanced the differentiating power. It is also interesting to note that these two TFs presented a moderate (r < 0.7) correlation with MTV (Figure A3) suggesting that they brought a valuable information in that respect.

These findings related to the use of TF are never been reported before using FDG-PET in a homogeneous population of PHEO. However, this work has some limitations including the single-center retrospective nature of this study which may weaken the final conclusion. The biological meaning of each TF remains unclear and it is therefore not straightforward to link these with a specific clinical outcome. However, provided that TFs are not correlated to either MTV or SUVmax, they can be considered as additional information that relies on the spatial heterogeneity of the tumor and/or genomics. The use of NGS assay for a limited number of our patients may have contributed to an underestimate of patients with mutation, most notably for the minor PHEO/PGL susceptibility genes recently identified. Similar to previously published studies, our cohort included a limited number of patients with mutations in a Cluster-1-related gene, due to the high prevalence of sporadic PHEO compared to PGLs. Additionally, somatic mutations could have an impact on tumoural FDG uptake, but have not been performed in this study. The presence of somatic cluster-1-related mutations may explain some cases of high FDG avidity observed in patients without determined germline mutations especially those with noradrenergic predomimant secretion [26]. Overall, our results would be prospectively validated on a larger group of patients with well-phenotyped PHEO including secretotype and genotyped by NGS at germline and somatic levels.

## 4. Materials and Methods

### 4.1. Patients

Consecutive patients evaluated for PHEO in our institution, from March 2007 to November 2015, were retrospectively included. All had an FDG-PET/CT and MIBG scan before surgery. Pre-operative plasma and/or urinary methoxyamine levels were measured. Methoxyamine levels/upper limits ratios were calculated and the predominant secretion was determined as the highest ratio. All resected tumors were histologically analyzed and evaluated with the predictive scoring systems PASS [27] and Ki-67 proliferation index. As recommended by the Endocrine society clinical practice guideline published in 2014 [1], PHEO/PGL genetic testing was systematically proposed to identify germline mutations in the major susceptibility genes (SDHB, SDHC, SDHD, VHL, RET, TMEM127, MAX) using Sanger sequencing and MLPA. Then, as recommended in the consensus statement published in 2017 [2], next-generation-sequencing (NGS)-based diagnostic was carried out for more recent patients (since 2016, *n* = 5 patients) with the panel described by Ben Aim et al. [28]. Informed consent was obtained for each patient before genetic analysis (with parental agreement obtained for minors) and surgery. In our institution ethical approval was waived by the local Ethics Committee of the University Hospital of Nantes in view of the retrospective nature of the study and all procedures being performed were part of the routine care.

### 4.2. FDG-PET/CT Acquisition Protocols

PET/CT scans were completed using a Discovery ST PET/CT system (GE Medical Systems, Milwaukee, WI, USA) for 18 patients and a Biograph mCT (Siemens Healthcare Molecular Imaging, Knoxville, TN, USA) for 31 patients. FDG was injected after 5 h of fasting and blood glucose measurement (median: 5.8 mmol/L). The injected activity was 5–7 MBq/kg for acquisitions performed on the Discovery ST PET system and 3 MBq/kg for acquisitions performed on the Biograph PET system. PET images were reconstructed using the algorithm provided by the manufacturer; OSEM without PSF correction, 2 iterations, 28 subsets and a Gaussian post-filtering of 5.45 mm FWHM for the Discovery ST, and OSEM with PSF correction and TOF information, 3 iterations, 21 subsets and a Gaussian post-filtering of 2.0 mm FWHM for the Biograph.

### 4.3. FDG-PET/CT Analysis

For visual analysis, PET/CT was considered positive if the intensity of the lesion uptake exceeded that of the liver, according to the criterion commonly used in the literature [29,30].

Several semi-quantitative parameters were derived from PET/CT images based on standard uptake value (SUV) for adrenal lesion and liver, and SUV ratios.

For TF analysis, adrenal lesions were automatically delineated on PET images using a consensus approach based on the simultaneous truth and performance level estimation (STAPLE) algorithm [31] using four different segmentations. Metabolic tumor volume (MTV) and the associated total lesion glycolysis (TLG) were subsequently derived for the most intense adrenal lesion. The total adrenal metabolic tumor volume (TMTV) and the whole-body total lesion glycolysis (wbTLG) excluding potential metastases were also calculated when patients presented several adrenal lesions.

Because two different PET systems were used in this study, the TFs were chosen based on their robustness in the context of the multicentric study [23,24,25] and as a function of the two discretization methods tested in this work (linear equalization using 64 bins and an absolute quantization using a fixed-width bins of 0.3 SUV). The implementation of TFs was conducted within the framework of the Image Biomarkers Standardization Initiative [32]. The grey level co-occurrence matrix (GLCM) and the grey level run length matrix (GLRLM) were calculated using a single matrix taking into account all 13 directions simultaneously with a one-voxel displacement. Finally, several TFs were also calculated based on the grey level size zone matrix (GLSZM). A minimal size of 64 voxels was required.

Five patients (5 lesions) were excluded from the TF analysis because of a too small lesion voxel size (*n* = 2), or adrenal lesions that were too complex to delineate which makes the segmentation unreliable (*n* = 2), and in one case, because of lost data.

### 4.4. Statistical Analysis

For statistical analysis, three groups of patients were considered: patients with mutations related to Cluster 1 and Cluster 2 (called “Cluster 1” and “Cluster 2”, respectively) and patients with no identified mutations (called “sporadic”).

The gold standard was based on histopathology analysis and biological follow up data. Sensitivity of imaging for PHEO diagnosis was calculated. The results of the PET-based visual analysis, semi-quantitative parameters and TF were analyzed according to different tumor-related parameters: lesion size, PASS score and its items, Ki67 index, secretory status and genetic status.

A Kruskal-Wallis test followed by a Dunn’s test (if required) controlled for family-wise error rate using the Hochberg’s progressive step down procedure to assess for the association between a continuous variable and a categorized variable (secretion and genetic status).

The correlation in-between quantitative PET-derived biomarkers was performed using the Spearman rank test adjusted for multiple comparison using the Benjamini-Hochberg approach.

Receiver operating characteristic (ROC) was performed for each PET parameter significantly associated with a categorized variable. The best cut-off was determined using the Youden index, and areas under the curve (AUC) between two parameters were compared using the DeLong method.

Subsequently, a model including a subset of selected TF was built to potentially improve the discrimination between patients with or without mutation when compared with the use of a single conventional PET parameter (MTV, TLG or SUVmax). First, only TF not correlated with MTV or SUVmax (r < 0.7) were selected to be included in the model. Then, a multiple logistic regression was conducted with those parameters together with MTV, TLG and SUVmax. Only the parameters selected in the previous step were kept to build the final model with a multiple logistic regression. A 4-fold validation with a subsampling that handled unbalanced classes using the SMOTE method was performed. The final model was compared to a model involving only MTV (or SUVmax) using the comparison of ROC curves.

Statistical analysis was conducted using R version 3.4.4.

## 5. Conclusions

FDG-PET/CT can be useful in the preoperative assessment of PHEO. In addition to improved initial lesion characterization, our results confirm the impact of genetic status on FDG uptake, based on semi-quantitative, metabolic volume parameters and TF analysis.

## Figures and Tables

**Table 1:** Patients and tumors characteristics.

Patients (*n* = 49)	Value [Range]
Sex ratio (F/M)	27/22
Median age (y)	49 [13–79]
Symptomatic (yes/no)	23/26
Secretion (yes/no/unknown)	44/2/3
Metanephrine predominant (*n*)	16
Normetanephrine predominant (*n*)	29
Similar normeta/metanephrine	1
Metastatic (no/yes)	47/2
**Genetic status (*n* = 49)**	
Cluster-1-related genes (SDHB, SDHD, VHL, FH)	4
Cluster-2-related genes (RET, NF1)	9
Sporadic	32
Indeterminate	4
**Pheochromocytomas (*n* = 52)**	**Value [Range]**
Unique (*n*)	46
Bilateral (*n*)	3
Median size (mm)	45 [17–300]
Median Ki-67	2 [1–30]
Median PASS score	4 [0–18]
MIBG (positive/negative)	39/13
FDG (positive/negative)	48/4

**Table 2:** Patients and tumors characteristics and FDG-PET results according to genetic status (after exclusion of 4 patients with indeterminate genetic status).

	Cluster-1-Related Genes	Cluster-2-Related Genes	Sporadic	*p*-Value
Number of patients	4	9	32	
Age (y)	36.5	43	49.5	0.38
Sex ratio (F/M)	2/2	4/5	17/15	0.98
Predominant production of metanephrine
(Patients without metanephrine excess
were removed from analysis)	0 (*n* = 2)	6 (*n* = 8)	9 (*n* = 32)	0.02
Number of PHEO	4	12	32	
Tumor size (mm)	39.5	34.5	56.5	0.003
PASS score	1.5	4	4	0.34
Ki67	2.5	1	2 (*n* = 28)	0.06

## References

[1] Lenders J., Duh Q., Eisenhofer G., Gimenez-Roqueplo A., Grebe S., Murad M., Naruse M., Pacak K., Young W. (2014). Pheochromocytoma and paraganglioma: An endocrine society clinical practice guideline. J. Clin. Endocrinol. Metab..

[2] Toledo R., Burnichon N., Cascon A., Benn D., Bayley J., Welander J., Tops C., Firth H., Dwight T., Ercolino T. (2017). Consensus Statement on next-generation-sequencing-based diagnostic testing of hereditary phaeochromocytomas and paragangliomas. Nat. Rev. Endocrinol..

[3] Van Berkel A., Pacak K., Lenders J. (2014). Should every patient diagnosed with a phaeochromocytoma have a ^123^I-MIBG scintigraphy?. Clin. Endocrinol. (Oxf.).

[4] Taïeb D., Sebag F., Hubbard J., Mundler O., Henry J., Conte-Devolx B. (2004). Does iodine-131 meta-iodobenzylguanidine (MIBG) scintigraphy have an impact on the management of sporadic and familial phaeochromocytoma?. Clin. Endocrinol. (Oxf.).

[5] Gimenez-Roqueplo A., Caumont-Prim A., Houzard C., Hignette C., Hernigou A., Halimi P., Niccoli P., Leboulleux S., Amar L., Borson-Chazot F. (2013). Imaging work-up for screening of paraganglioma and pheochromocytoma in SDHx mutation carriers: A multicenter prospective study from the PGL.EVA Investigators. J. Clin. Endocrinol. Metab..

[6] Amodru V., Guerin C., Delcourt S., Romanet P., Loundou A., Viana B., Brue T., Castinetti F., Sebag F., Pacak K. (2018). Quantitative 18F-DOPA PET/CT in pheochromocytoma: The relationship between tumor secretion and its biochemical phenotype. Eur. J. Nucl. Med. Mol. Imaging.

[7] Luster M., Karges W., Zeich K., Pauls S., Verburg F.A., Dralle H., Glatting G., Buck A., Solbach C., Neumaier B. (2010). Clinical value of 18F-fluorodihydroxyphenylalanine positron emission tomography/computed tomography (18F-DOPA PET/CT) for detecting pheochromocytoma. Eur. J. Nucl. Med. Mol. Imaging.

[8] Moreau A., Giraudet A., Kryza D., Borson-Chazot F., Bournaud C., Mognetti T., Lifante J., Combemale P., Giammarile F., Houzard C. (2017). Quantitative analysis of normal and pathologic adrenal glands with 18F-FDOPA PET/CT: Focus on pheochromocytomas. Nucl. Med. Commun..

[9] Timmers H., Kozupa A., Chen C., Carrasquillo J., Ling A., Eisenhofer G., Adams K., Solis D., Lenders J., Pacak K. (2007). Superiority of fluorodeoxyglucose positron emission tomography to other functional imaging techniques in the evaluation of metastatic SDHB-associated pheochromocytoma and paraganglioma. J. Clin. Oncol..

[10] Taïeb D., Hicks R., Hindié E., Guillet B., Avram A., Ghedini P., Timmers H., Scott A., Elojeimy S., Rubello D. (2019). European Association of Nuclear Medicine Practice Guideline/Society of Nuclear Medicine and Molecular Imaging Procedure Standard 2019 for radionuclide imaging of phaeochromocytoma and paraganglioma. Eur. J. Nucl. Med. Mol. Imaging.

[11] Tiwari A., Shah N., Sarathi V., Malhotra G., Bakshi G., Prakash G., Khadilkar K., Pandit R., Lila A., Bandgar T. (2017). Genetic status determines 18 F-FDG uptake in pheochromocytoma/paraganglioma. J. Med. Imaging Radiat. Oncol..

[12] Van Berkel A., Rao J., Kusters B., Demir T., Visser E., Mensenkamp A., van der Laak J., Oosterwijk E., Lenders J., Sweep F. (2014). Correlation between in vivo 18F-FDG PET and immunohistochemical markers of glucose uptake and metabolism in pheochromocytoma and paraganglioma. J. Nucl. Med..

[13] Timmers H., Chen C., Carrasquillo J., Whatley M., Ling A., Eisenhofer G., King K., Rao J., Wesley R., Adams K. (2012). Staging and functional characterization of pheochromocytoma and paraganglioma by 18F-fluorodeoxyglucose (18F-FDG) positron emission tomography. J. Natl. Cancer Inst..

[14] Taïeb D., Sebag F., Barlier A., Tessonnier L., Palazzo F., Morange I., Niccoli-Sire P., Fakhry N., De Micco C., Cammilleri S. (2009). 18F-FDG avidity of pheochromocytomas and paragangliomas: A new molecular imaging signature?. J. Nucl. Med..

[15] Bozkurt M., Virgolini I., Balogova S., Beheshti M., Rubello D., Decristoforo C., Ambrosini V., Kjaer A., Delgado-Bolton R., Kunikowska J. (2017). Guideline for PET/CT imaging of neuroendocrine neoplasms with 68Ga-DOTA-conjugated somatostatin receptor targeting peptides and 18F-DOPA. Eur. J. Nucl. Med. Mol. Imaging.

[16] Lambin P., Rios-Velazquez E., Leijenaar R., Carvalho S., van Stiphout R., Granton P., Zegers C., Gillies R., Boellard R., Dekker A. (2012). Radiomics: Extracting more information from medical images using advanced feature analysis. Eur. J. Cancer.

[17] Hatt M., Tixier F., Pierce L., Kinahan P., Le Rest C., Visvikis D. (2017). Characterization of PET/CT images using texture analysis: The past, the present… any future?. Eur. J. Nucl. Med. Mol. Imaging.

[18] Zhang G., Shi B., Sun H., Jin Z., Xue H. (2017). Differentiating pheochromocytoma from lipid-poor adrenocortical adenoma by CT texture analysis: Feasibility study. Abdom. Radiol. (N. Y.).

[19] Nakajo M., Jinguji M., Nakajo M., Shinaji T., Nakabeppu Y., Fukukura Y., Yoshiura T. (2017). Texture analysis of FDG PET/CT for differentiating between FDG-avid benign and metastatic adrenal tumors: Efficacy of combining SUV and texture parameters. Abdom. Radiol. (N. Y.).

[20] Plouin P., Amar L., Dekkers O., Fassnacht M., Gimenez-Roqueplo A., Lenders J., Lussey-Lepoutre C., Steichen O., Amar L., Dekkers O. (2016). European Society of Endocrinology Clinical Practice Guideline for long-term follow-up of patients operated on for a phaeochromocytoma or a paraganglioma. Eur. J. Endocrinol..

[21] Tessonnier L., Ansquer C., Bournaud C., Sebag F., Mirallié E., Lifante J., Palazzo F., Morange I., Drui D., de la Foucardère C. (2013). (18)F-FDG uptake at initial staging of the adrenocortical cancers: A diagnostic tool but not of prognostic value. World J. Surg..

[22] Favier J., Brière J., Burnichon N., Rivière J., Vescovo L., Benit P., Giscos-Douriez I., De Reyniès A., Bertherat J., Badoual C. (2009). The Warburg effect is genetically determined in inherited pheochromocytomas. PLoS ONE.

[23] Bailly C., Bodet-Milin C., Couespel S., Necib H., Kraeber-Bodéré F., Ansquer C., Carlier T. (2016). Revisiting the Robustness of PET-Based Textural Features in the Context of Multi-Centric Trials. PLoS ONE.

[24] Desseroit M., Tixier F., Weber W., Siegel B., Cheze Le Rest C., Visvikis D., Hatt M. (2017). Reliability of PET/CT Shape and Heterogeneity Features in Functional and Morphologic Components of Non-Small Cell Lung Cancer Tumors: A Repeatability Analysis in a Prospective Multicenter Cohort. J. Nucl. Med..

[25] Van Velden F., Kramer G., Frings V., Nissen I., Mulder E., de Langen A., Hoekstra O., Smit E., Boellaard R. (2016). Repeatability of Radiomic Features in Non-Small-Cell Lung Cancer [(18)F]FDG-PET/CT Studies: Impact of Reconstruction and Delineation. Mol. Imaging Biol..

[26] Eisenhofer G., Klink B., Richter S., Lenders J., Robledo M. (2017). Metabologenomics of phaeochromocytoma and paraganglioma: An integrated approach for personalised biochemical and genetic testing. Clin. Biochem. Rev..

[27] Mlika M., Kourda N., Zorgati M., Bahri S., Ben Ammar S., Zermani R. (2013). Prognostic value of Pheochromocytoma of the Adrenal Gland Scaled Score (Pass score) tests to separate benign from malignant neoplasms. Tunis. Med..

[28] Ben Aim L., Pigny P., Castro-Vega L., Buffet A., Amar L., Bertherat J., Drui D., Guilhem I., Baudin E., Lussey-Lepoutre C. (2019). Targeted next-generation sequencing detects rare genetic events in pheochromocytoma and paraganglioma. J. Med. Genet..

[29] Blake M., Slattery J., Kalra M., Halpern E., Fischman A., Mueller P., Boland G. (2006). Adrenal lesions: Characterization with fused PET/CT image in patients with proved or suspected malignancy–initial experience. Radiology.

[30] Ansquer C., Scigliano S., Mirallié E., Taïeb D., Brunaud L., Sebag F., Leux C., Drui D., Dupas B., Renaudin K. (2010). 18F-FDG PET/CT in the characterization and surgical decision concerning adrenal masses: A prospective multicentre evaluation. Eur. J. Nucl. Med. Mol. Imaging.

[31] Warfield S., Zou K., Wells W. (2004). Simultaneous truth and performance level estimation (STAPLE): An algorithm for the validation of image segmentation. IEEE Trans. Med. Imaging.

[32] Zwanenburg A., Vallières M., Abdalah M., Aerts H., Andrearczyk V., Apte A., Ashrafinia S., Bakas S., Beukinga R., Boellaard R. (2020). The Image Biomarker Standardization Initiative: Standardized Quantitative Radiomics for High-Throughput Image-based Phenotyping. Radiology.

